# “I've got plenty of energy when I’m doing something I want to do”: applying self-determination theory to exercise motivation in people with prostate cancer

**DOI:** 10.1007/s00520-025-09410-z

**Published:** 2025-04-03

**Authors:** Harriet Naismith, Haryana M. Dhillon, Julia Hunter, Renee Bultijnck, Andrew Kneebone, George Hruby, Thomas Eade, Lisa Parker, Rachel Campbell, Jasmine Yee

**Affiliations:** 1 School of Psychology, University of Sydney, Sydney, NSW Australia; 2 Psycho-Oncology Cooperative Research Group, School of Psychology, University of Sydney, Sydney, NSW Australia; 3 Centre for Medical Psychology and Evidence-Based Decision-Making, School of Psychology, University of Sydney, Sydney, NSW Australia; 4 Dept of Radiation Oncology, Northern Sydney Cancer Centre, Royal North Shore Hospital, St Leonards, Sydney, NSW Australia; 5 Department of Human Structure and Repair, Ghent University, Ghent, Belgium; 6 Northern Clinical School, Faculty of Medicine and Health, University of Sydney, Sydney, NSW Australia; 7 Sydney School of Health Sciences, Faculty of Medicine and Health, University of Sydney, Sydney, NSW Australia

**Keywords:** Prostate cancer, Androgen deprivation therapy, Exercise, Self-determination theory, Qualitative research

## Abstract

**Purpose:**

Androgen deprivation therapy (ADT) for prostate cancer adversely affects quality of life. Whilst exercise is effective for ameliorating many side effects, most people are inactive, with lack of motivation a key barrier. Self-determination theory (SDT) specifies the quality, rather than quantity, of motivation as essential for optimal engagement. We aimed to explore exercise motivation in men on ADT through the theoretical lens of SDT.

**Methods:**

As part of a mixed-method longitudinal study, semi-structured interviews exploring exercise behaviour and perceptions, were conducted with people receiving ADT for prostate cancer. Thematic analysis identified motivation themes aligned with SDT concepts.

**Results:**

Twenty-four men participated (median age 74 years; ECOG 0: 92%, metastatic: 29%). We identified two key themes: (1) type of exercise motivation and (2) use of need-supportive techniques in exercise environments. Motivations ranged from intrinsic (for enjoyment) to external (compliance with other’s expectations). Key strategies to support psychological needs included offering choice in exercise programming, providing meaningful rationales for exercise, tailored guidance from exercise professionals, and social support.

**Conclusions:**

This SDT-grounded study provides insights into motivations driving exercise in people receiving ADT and how social and healthcare contexts influence these motivations. The study underscores the importance of considering exercise motivation when discussing, referring, and designing tailored exercise interventions to ensure they are need-supportive to optimise engagement.

Implications for cancer survivors.

This study highlights the importance of exercise interventions that are supportive of psychological needs. Incorporating need-supportive strategies may enhance exercise participation and improve physical and psychosocial outcomes for men receiving ADT.

**Supplementary Information:**

The online version contains supplementary material available at 10.1007/s00520-025-09410-z.

## Introduction

Androgen deprivation therapy (ADT) is a cornerstone of treatment for men with high-risk and metastatic prostate cancer [1]. Although ADT improves clinical outcomes by reducing circulating androgens, it also causes side effects that can negatively impact quality of life through metabolic, sexual, cognitive, skeletal and cardiovascular changes [2]. Chronic or clinically significant fatigue is reported by ~ 40% of those on long-term ADT, interfering with daily functioning [3]. Additionally, those receiving ADT report greater levels of depression, poorer body image and reduced quality of life [4].

Exercise is a well-established strategy to improve outcomes such as fatigue, anxiety, physical function and quality of life (QOL) in prostate cancer survivors [5–7]. Epidemiological evidence suggests physical activity can extend survival, lower risk of recurrence and reduce cancer-specific mortality for people with prostate cancer [8, 9]. Despite these benefits, only 12% of Australian people with prostate cancer meet recommended exercise guidelines of ≥ 150 min of moderate-intensity or ≥ 75 min of vigorous-intensity aerobic exercise and 2–3 resistance sessions per week [10, 11]. This low prevalence of exercise means many miss out on the benefits of exercise in mitigating ADT-related side effects and improving clinical outcomes.

Motivation is crucial for initiating and maintaining exercise behaviour [12]. While fostering exercise motivation is a major challenge in any population, it is especially difficult in cancer settings due to treatment-related debilitation [13]. Reduced motivation is a well-documented barrier to exercise engagement among people with prostate cancer, including those receiving ADT [14–16]. In a qualitative study, people with prostate cancer on ADT identified reduced motivation as a significant barrier to physical activity [17]. In contrast, those not receiving ADT did not express diminished motivation as a barrier. This suggests that reduced motivation may be associated with the effects of ADT on physical capabilities and energy levels, highlighting the need for further exploration of exercise motivation in this population.

**Fig. 1:**
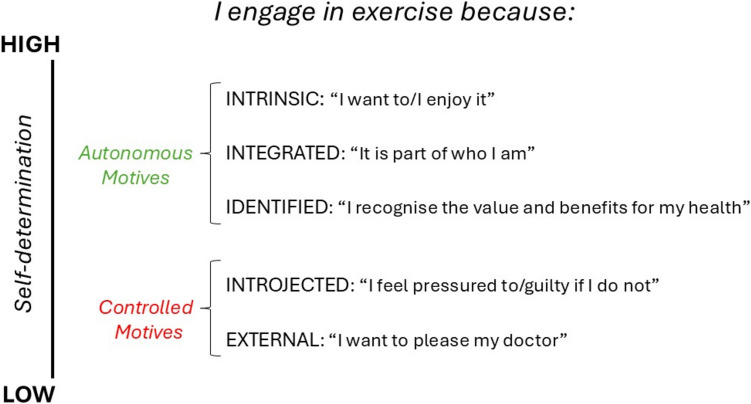
The motivation continuation according to self-determination theory [18]. Adapted from Quested et al. 2021 [19]

Self-determination theory (SDT) is a broad theory useful to understand human motivation for health behaviours, including exercise initiation and maintenance. SDT focuses on the quality rather than quantity of motivation, differentiating between more autonomous or voluntary and more controlled or pressured types of motivation [18]. Autonomous motivation is considered the optimal form of motivation consisting of three subtypes (Fig. 1): *intrinsic motivation* refers to participation in exercise for its own sake because it is inherently enjoyable or optimally challenging; *integrated motivation* refers to participation in exercise because of its alignment with one’s identity or broader set of life values; and *identified motivation* refers to engaging in exercise because of recognition of its value and worth [18]. In contrast, controlled motivation is a pressured, suboptimal form consisting of two subtypes (Fig. 1): *introjected motivation* resulting from internal pressures such as guilt or fear and *external motivation* arising from external pressure to exercise (e.g. from a doctor or spouse). These different types of motivation are conceptualised on a continuum from highly controlled (non-self-determined) to autonomous (highly self-determined) [18].

Extensive evidence links autonomous motivation with positive outcomes, including higher exercise levels and greater exercise persistence, engagement and enjoyment [20, 21]. Research demonstrates positive associations between autonomous motivation and higher physical activity levels in breast [22] and mixed cancer populations [23]. In contrast, controlled motivation is associated with poorer exercise outcomes such as lower exercise levels, boredom, disengagement and reduced adherence over time [20, 21, 24, 25].

SDT further delineates how social-contextual factors can either support or thwart motivation through the satisfaction of basic psychological needs for autonomy, competence and relatedness [25]. Need-supportive environments fulfil these psychological needs by the person delivering an exercise intervention: (i) providing meaningful choice, a clear rationale, and acknowledging the individuals’ perspective (supporting autonomy); (ii) ensuring exercise is optimally challenging with guidance and structure, and negative feedback is avoided (supporting competence); and (iii) being warm, empathic and genuinely interested in supporting the individual to exercise (supporting relatedness). Meta-analyses of intervention studies using need-supportive behaviour change techniques grounded in SDT have shown positive effects on autonomous motivation and physical activity [21, 26].

Despite the potential of SDT to enhance understanding of what drives people with prostate cancer on ADT to (dis)engage in exercise behaviour, no studies have used this theoretical framework to examine exercise motivation in this population. Whilst quantitative research has generated compelling evidence regarding exercise motivation and associated outcomes, a qualitative approach would enable more comprehensive exploration of unique motivational drivers in this population. Using SDT as a theoretical lens, we aimed to use qualitative methods to gain in-depth understanding of the types of exercise motivation reported in this population and to explore perceptions of what need-supportive strategies are used within this populations’ social and healthcare contexts.

## Method

### Study design

This qualitative study is a sub-study of a mixed-method longitudinal project exploring factors impacting uptake and adherence to exercise in people receiving ADT. Participants were followed for 12 months, completing questionnaires and interviews at baseline, then 3-monthly. Participants were also offered referral to existing community-based exercise programs following enrolment.

Here, we report analysis of baseline qualitative data. This analysis used deductive qualitative methods of thematic analysis [27] to explore exercise motivation from the perspective of people receiving ADT treatment for prostate cancer. This study was embedded within a realist/essentialist paradigm, assuming experience and meaning are shared in a straightforward way, which is suitable for exploring the construct of motivation [27].

### Participants

Eligible participants were required to have been diagnosed with prostate cancer, currently receiving ADT as part of their treatment and be 18 years or older. Participants were excluded if they could not communicate in English, had health risk factors precluding exercise or logistic, health or cognitive concerns preventing adherence to study procedures. A convenience sample was used, and all participants enrolled in the longitudinal mixed-methods study were invited to complete a baseline interview. Ethical approval was obtained through the Northern Sydney Local Health District (HREC 2019/ETH03959).

### Procedure

Recruitment was conducted through one metropolitan Sydney hospital. Eligible participants were informed about the study by the Prostate Cancer Nurse Coordinator or their Radiation Oncologist. Potential participants were contacted by a researcher either in person at the hospital or via email to discuss study requirements and provided a participant information sheet. Written informed consent was obtained prior to any study procedures.

### Data collection

#### Quantitative data collection

Participants completed a demographic questionnaire capturing education level, language spoken at home, Indigenous status, country of birth and living arrangements. Clinical characteristics including age, smoking status, comorbidities, Eastern Cooperative Oncology Group (ECOG) performance status score, date of cancer diagnosis, cancer staging and treatment details were obtained from medical records. Exercise behaviour was captured using the modified Godin Leisure Time Exercise Questionnaire (GLTEQ) where participants reported frequency and average duration of mild, moderate, vigorous and resistance exercise performed over the past 7 days [28].

#### Qualitative data collection

A semi-structured interview guide was developed to address the aims of the longitudinal mixed-method study (Supplementary File 1) and was iteratively revised during data collection. Individual semi-structured interviews were conducted by a member of the research team (JY). JY (female, BSc/PhD) is an academic exercise physiologist with expert knowledge in exercise oncology and qualitative methods, working as a postdoctoral research fellow under the mentorship of author HD. Before baseline interviews, JY had briefly spoken to participants about their background, the research project, and exercise but had no pre-existing relationship. Participants were briefed on the aims of the study to explore exercise engagement in people receiving ADT.

During the interview, participants were asked about their exercise behaviours and motivation to exercise, followed by a more focused discussion around challenges to engaging in and maintaining exercise, as well as perceptions regarding healthcare professionals’ role in supporting exercise. Interviews were conducted with participants either via telephone or in person at the hospital. Each interview was audio recorded, with notes taken, and later transcribed with the assistance of automatic transcription software (Trint [29]). To ensure the integrity and quality of transcription, each transcript was reviewed and manually corrected by the research team to address any errors introduced by the software. Only the participant and interviewer were present during interviews.

#### Data analysis

Demographic and clinical data were analysed descriptively with IBM SPSS Statistics (Version 27). Baseline GLTEQ responses were compared with Clinical Oncology Society of Australia (COSA) exercise guidelines (≥ 150 min of moderate-intensity or ≥ 75 min of vigorous-intensity aerobic exercise and 2–3 resistance sessions per week) to determine if participants met exercise recommendations.

Interview data were thematically analysed [30]. After review of themes identified, we employed a deductive analysis approach by applying SDT as a conceptual lens to explore exercise motivation and specific SDT concepts (i.e. motivation regulations, need-supportive/thwarting strategies) reported by participants.

All data were imported into NVivo12 [31]. Two researchers (HN and RC) cross-coded the initial five transcripts. Codes were pre-defined by SDT or derived from the data, as appropriate. A working analytical framework was developed, which HN applied to all transcripts. The data were summarised into a matrix in Microsoft Excel, organised by participant cases and coded categories, which was analysed to identify important themes, relationships and theoretical concepts. Reporting adhered to the 32-item consolidated criteria for qualitative reporting [32] (COREQ; Supplementary File 2).

Alongside illustrative quotes, participant characteristics (study ID, age and whether they met exercise guidelines or not (GL:AO = meeting aerobic exercise guidelines; GL:N = not meeting exercise guidelines; GL:RO = meeting resistance exercise guidelines; GL:Y = meeting aerobic and resistance guidelines) are reported.

## Results

Of the 50 participants enrolled in the longitudinal mixed methods study, 34 completed baseline semi-structured interviews. Reasons for non-participation in the interview included the following: (i) not contactable (*n* = 11); (ii) unavailable due to work or travel commitments (*n* = 3), (iii) hospitalisation (*n* = 1); and (iv) ADT treatment at another site (*n* = 1). Thematic saturation was achieved after analysis of 21 interviews and confirmed by a further three interviews, resulting in a total of 24 interviews analysed and included here. Median interview duration was 42 min (range 21–63).

Table 1 presents participant characteristics. Participant median age was 74 years (range 45–88). Most lived with their spouse (79%), were born in Australia (50%), spoke English at home (92%) and had completed tertiary education (58%). The median time since prostate cancer diagnosis was 2.5 years (range 0–21 years), with 29% having metastatic prostate cancer. The majority were not meeting exercise guidelines (87%).

**Table 1:** Participant characteristics (*n* = 24)

Characteristic	*n* (%)
Age (years), median (range)	74 (45–88)
Living alone	5 (21)
Country of birth	
Australia	12 (50)
New Zealand	1 (4)
Europe	8 (33)
Asia	3 (13)
Aboriginal or Torres Strait Islander	0 (0.0)
English spoken at home	22 (92)
Education level	
Did not complete high school	1 (4)
Completed high school	8 (33)
University	14 (58)
TAFE/Trade	1 (4)
Smoking status	
Never	13 (54)
Former	6 (25)
Current	2 (8)
Missing	3 (12)
Number of Comorbidities	
0	5 (21)
1	10 (42)
2	6 (25)
3 +	3 (13)
ECOG Performance status	
0	22 (92)
1	1 (4)
2	1 (4)
Time (years) since prostate cancer diagnosis	
0–5	13 (54)
6–10	4 (17)
11–15	5 (21)
16 +	2 (8)
Metastatic prostate cancer	7 (29)
Previous treatment	
None	9 (38)
Radiotherapy	3 (13)
Prostatectomy	7 (29)
Radiotherapy & prostatectomy	5 (21)
Concurrent treatment with ADT	
Radiotherapy	14 (58)
None	10 (42)
History of other cancer	3 (13)
MVPA $\ge$ 150 min/week	5 (21)
Resistance exercise $\ge$ 2 sessions/week	10 (42)
Meeting COSA exercise guidelines	3 (13)

*ADT* androgen deprivation therapy, *ECOG* Eastern Cooperative Oncology Group, *MVPA* moderate-vigorous physical activity, *COSA* Clinical Oncology Society of Australia

### Themes

Two themes encompassing core concepts of SDT were identified from the data: (1) type of exercise motivation and (2) use of need-supportive techniques in exercise environments.

#### Theme 1: Types of exercise motivation

Theme 1 focused on the types of exercise motivation underlying participants’ exercise engagement as per the SDT motivation continuum. Additional illustrative quotes are provided in Table 2.

**Table 2:** Illustrative quotations for Theme 1: Type of exercise motivation

Subtheme	Code	Quotation
Intrinsic motivation	Innate enjoyment Inherently interesting	*I love to do five days of walk and two days of bike riding. That's what I want to do.* (P24, 78y, GL:AO) *It’s like if you are walking around the park and you see the owls that are there, you are looking for something and it is good.* (P02, 91y, GL:N)
Integrated motivation	Exercise identity	*The reason is in my daily life, in this stage, I wanted physical activity to be part of my daily life. I mean, it has to be part of my life because I got to be physically active.* (P24, 78y, GL:AO)
Identified motivation	Valued physical benefits Cancer-specific Valued emotional benefits Valued role functioning Valued cognitive benefits	*Good cardiovascular fitness and muscle strength means you’re overall healthier.* (P27, 69y GL:AO) *The PSA [prostate-specific antigen] increase seemed to slow down as well, you know, being fit makes you better.* (P14, 70y, GL:AO) *I think is the main thing that you don’t sort of sit around and mope about your condition at all … you know, taking that attitude, that you’ve got, got to do something positive and exercising is as good as anything.* (P05, 84y, GL:RO) *The endorphin generation that comes with the exercise makes me feel good.* (P19, 89y, GL:N) *I want to be fit for my daughter … I don’t want her growing up with a sick father.* (P15, 47y, GL:RO) *I’m hoping that exercise keeps my mental faculties going as well so I can continue on to be engaged.* (P21, 65y, GL:N)
Introjected motivation	Internal pressure Internalised social norms Guilt over inactivity Fear	*I will sometimes have to force myself to do it [exercise], but I will make sure that I bloody well do it.* (P01, 68y, GL:N) *I think I’m too big, too big to be weak.* (P15, 47y, GL:RO) *If I don't walk and look after myself, there will be recognition that I won't be you know, I won't be well, be fit.* (P16, 66y, GL:N) *I realise I’ve been watching sport for an hour, seeing people run around, and I think Christ, I’d best get up and at least walk around the room.* (P05, 85y, GL:RO) *I got a boot in the backside when I got those results back about osteopenia … so that sort of thing is motivational and on the scarier side of things.* (P17, 78y, GL:AO)
External motivation	Comply with external demands	*Having a personal trainer like that sort of was very good because somebody telling me what to do and I'd just do it.* (P20, 81y, GL:N) *Because my wife has been pushing me to do ‘em [resistance exercises]. That’s alright, she’s my own motivator.* (P04, 71y, GL:N)

*GL:AO* meeting aerobic exercise guidelines, *GL:N* not meeting exercise guidelines, *GL:RO* meeting resistance exercise guidelines, *GL:Y* meeting combined aerobic and resistance guidelines

##### Intrinsic

Several participants exercised for enjoyment and pleasure. These participants wanted to exercise and did so of their own volition. Some described exercise as inherently satisfying and an opportunity to spend time outdoors in nature.

*I know that if I go walking with my bushwalking friend, I mean we can walk for six or eight hours ... I've got plenty of energy when I'm doing something that I want to do.* (P07, 70y, GL:Y)

It was common for those with intrinsic exercise motivation to have prior exercise experience. These participants prioritised exercise and found solutions to overcome barriers to exercise, such as injuries. Some participants with intrinsic exercise motivation also reported enjoying and engaging in other healthy lifestyle behaviours such as maintaining a healthy diet.

##### Integrated

 A few participants described that exercise overlapped with core values and sense of self, with exercise forming part of their identity.

*It's just part of my, my psyche.* (P08, 84y, GL:N)

##### Identified

Many exercised because they valued the experience or anticipated benefits. Participants frequently reported exercising to improve and prolong their quality of life.

*My motivation for physical activity … is a general sense of well-being to maintain the quality of life. And that’s the most important thing to me, to live longer, live healthily, well, mentally, physically. Quality of life, that’s what I’m after.* (P13, 73y, GL:Y)

Many also acknowledged general health benefits, including lowering blood pressure and improving sleep. Some noted age-specific benefits, such as maintaining mobility. One reported exercising to prevent ADT-related side effects.

Many also reported exercising to experience *emotional* benefits, commonly exercising to ‘feel good’. A few participants indicated exercise relieved stress or anxiety. For some, exercise gave them mental strength to remain positive while living with cancer, also enhancing their self-concept and confidence. Those with prior exercise experience more commonly endorsed the personal value of exercise, encouraged by physical and emotional benefits experienced previously when exercising.

*I suffer from anxiety as well, so you get less anxiety while you're exercising. So it helps you all round I think.* (P14, 70y, GL:AO)

A few participants exercised to maintain their ability to carry out their usual activities including caring responsibilities or engaging in recreational activities. Retired participants valued exercise to help maintain their independence in daily activities. Some participants valued the *cognitive* benefits of exercise, such as improved concentration or mental clarity.

Introjected. Many exercised because they felt they ‘should’, ‘must’ or ‘have to’. A few described an internalised, pressuring voice, influencing their exercise behaviour. Some exercised to uphold internalised masculinity norms to be strong and ‘buff’. Many exercised due to a perceived obligation to maintain an acceptable weight, with some viewing being overweight as a character flaw. A few participants described their self-worth as contingent on exercising. Experiencing guilt over inactivity also drove many participants to engage in exercise. However, this engagement was often short-lived. Participants with introjected motivation often reported barriers to exercise including, feeling old, lack of time due to work or other commitments, and poor weather.

*Both of us [participant and wife] know we should be doing the other exercises and both of us every now and again get guilty and do it and then don't.* (P17, 78y, GL:AO)

##### External

Some participants exercised to comply with demands of others, including partners or gym instructors. Often participants expressing extrinsic exercise motivation attributed their inactivity to being lazy. Some stated they would only exercise in a gym environment with a personal trainer to hold them accountable.

*If you want to do it [exercise] properly it's probably better to do it in a gym or somewhere, where someone has got the whip out.* (P22, 82y, GL:N)

#### Theme 2: Use of need-supportive techniques in exercise environments

Theme 2 focuses on strategies used within the social and healthcare context that influenced participants’ basic psychological needs. See Table 3 for further illustrative quotations. A summary of strategies and examples for promoting autonomous motivation is provided in Table 4.

**Table 3:** Illustrative quotations for Theme 2: Use of need-supportive techniques in social and healthcare context

Subtheme	Quotation
Providing choice	*Oh if I’m home, I’d do that [resistance exercise], that’s not a problem. I’d rather sit out on the balcony and hear the birds and look at the trees and smell the fresh air than in a gym doing things like that*. (P10, 74y, GL:RO) *I’d rather be doing [the resistance program] at home so I’m sort of a bit more flexible.* (P16, 67y, GL:N)
Providing a meaningful rationale	*“You read about, you hear them talking on the radio, you know, like people should be doing this much walking every day, that much walking every day. I don't really know what benefit it does.”* (P22, 82y, GL:N) *“And that's perhaps the best advice. The best way for me to get advice about exercise is to be told. "Here are the benefits, you're not in a good place at the moment. You can do something about it". So I will. Easy.”* (P28, 76y, GL:N)
Structured help and guidance	*He's [gym instructor] really guided me as to what I should be doing and introducing new elements and talking to me, it's virtually individual supervision. I mean, I think it's tremendous.* (P12, 86y, GL:N) *I mentioned he [personal trainer] was very good and targeted us and our age and all our problems.* (P17, 78y, GL:AO) *I have been doing these exercises with the physiotherapist … they check to make sure I've got the technique correct and then they go away and set out the exercises that we were doing so I have a written documentation that I can refer back to, to make sure I stay on track.* (P26, 80y, GL:N)
Authentic interest and encouragement	*You know, I go training or whatever, she [wife] always supports me doing it.* (P14, 70y, GL:RO) *I’ve got all this support network around the surrounding that care for me and I know they care for me, and it's a feeling I have 24/7.* (P13, 73y, GL:Y)

*GL:AO* meeting aerobic exercise guidelines, *GL:N* not meeting exercise guidelines, *GL:RO* meeting resistance exercise guidelines, *GL:Y* meeting combined aerobic and resistance guidelines

**Table 4:** Need-supportive strategies and practical examples for promoting autonomous (optimal) motivation for exercise in people receiving androgen deprivation therapy (ADT)

Need-supportive strategy	Example
Providing choice *Offer flexibility in exercise options such as choosing between home- or gym-based, indoor or outdoor, and group or independent exercise.*	Emphasise that the choice of exercise location is based on personal preference, with benefits achievable both at home or in a gym.
Providing a meaningful rationale for exercise *Alongside recommending exercise, provide personalised explanations of its benefits, particularly for those receiving ADT.*	Explain how exercise can counteract side effects specifically associated with ADT, such as improving body composition (reducing fat and increasing muscle) and managing fatigue. The explanation should be customised based on which side effects are most bothersome to the individual.
Structured help and guidance *Tailor exercise programs and support with consideration of factors such as age, pre-existing conditions, and exercise history.*	Offer detailed exercise instructions and demonstrations for those new to exercise, along with ongoing and structured support. Refer to an exercise professional (e.g. exercise physiologist) if further expertise is needed.
Authentic interest and encouragement *Build rapport and encourage social support where appropriate.*	Acknowledge the individual’s perspective and encourage asking of questions. If appropriate, involve family or friends in discussions of exercise to ensure they understand the role of exercise in managing ADT side effects.

##### Providing choice

Exercise professionals supported autonomy by offering choice over exercise characteristics (e.g. choice between home- or gym-based exercise programs). Most participants opted for home-based programs, preferring to exercise independently, outdoors, or have a flexible exercise regimen. Some participants chose to exercise at home to avoid feeling pressured into exercises they did not want to do or in an environment they find uncomfortable.

*And, as I said I'm not a gym person, I just don't want to go to a gym. *(P10, 74y, GL:RO)

A few opted to exercise at a gym because they valued variety in their exercise routine.

##### Providing a meaningful rationale

Most participants stated primary care physicians were best placed to provide personally meaningful reasons to exercise, ideally during face-to-face consultations. However, many reported healthcare professionals did not discuss exercise during appointments. Some indicated consultations were too short for exercise to be addressed. Most only had brief or general conversations about exercise with healthcare professionals. Participants reported these discussions often lacked explanation of why exercising was important.

*You’re simply just told to do it [exercise]. I mean, just simply do it. You’re not told the reason why and what benefits are associated with what you’re actually doing.* (P21, 65y, GL:N)

As a result, many participants were not aware of the benefits of exercise, either in general or specifically related to ADT treatment. Only a minority were aware of the positive impact of exercise in managing ADT side-effects.

##### Structured help and guidance

Exercise competence was bolstered by exercise instructors who helped individuals feel capable in their ability to complete exercises. Many participants described the importance of being shown how to do a specific exercise and tailoring instructions to each individual’s capabilities. Participants felt more confident when exercise professionals took factors such as age or pre-existing injuries into account when designing exercise programs. Participants with little prior exercise experience expressed a strong desire for guidance from exercise professionals. Some indicated a preference for intensive, structured support during the initial stages of learning to exercise. Many also expressed a desire for ongoing guidance to ensure they continued to feel confident when exercising; however, this was less important for participants with prior exercise experience.

*I just want someone to teach me what I should be doing. I know I should be doing it, and I will do it if I know that I’m capable and know what to do.* (P23, 78y, GL:N)

##### Authentic interest and encouragement

Almost all participants discussed the importance of receiving support to exercise from their social network. Many experienced support and positive encouragement to exercise from their partners and children. Participants reported a sense of connectedness when their children were also passionate about health and fitness.

A few participants also reported the importance of experiencing a sense of connection with exercise professionals. However, they conveyed divergent feelings about needing social connection when exercising. One participant’s connection with his gym instructor encouraged him to exercise.

*The young physio guy had a great rapport with me and that helped motivate me.* (P09, 78y, GL:RO)

Yet, some participants conveyed a ‘leave it to me’ attitude, expressing no desire for connectedness with exercise professionals.

*I don't need anybody to either encourage me or make me do exercise. *(P07, 70y, GL:Y)

## Discussion

In this study, we used SDT as a theoretical lens to explore exercise motivation among people with prostate cancer on ADT. Our findings provide novel insights into the motivation underlying exercise engagement and the role of the social and healthcare context in influencing exercise motivation by supporting or thwarting basic psychological needs.

We demonstrated people on ADT were motivated to engage in exercise for an array of reasons, spanning the full spectrum of motivation as posited by SDT. Encouragingly, more autonomous forms of motivation underpinned many participants’ exercise engagement, with most driven by the physical, emotional, health and overall quality of life benefits (identified motivation). However, several participants reported suboptimal, controlled types of exercise motivation. The desire to conform to social masculinity norms is consistent with previous research suggesting some people on ADT are motivated to exercise to counter loss of muscle mass and perceived loss of masculinity [14, 17]. Externally motivated participants exercised to meet other’s expectations (e.g. personal trainers, spouse), while these controlled motives can prompt short-term exercise behaviour, it is unlikely to be sustained over time [20, 33].

Although suboptimal forms of motivation can hinder long-term engagement in exercise, SDT positions motivation as dynamic and malleable [18]. Research shows that people receiving ADT who participated in a clinical pathway inclusive of exercise, shifted towards more autonomous motivation once engaged in the pathway [34]. Healthcare professionals play a crucial role and can help individuals develop more autonomous motives by using strategies supporting autonomy, competence, and relatedness, enabling them to transition along the motivation continuum [18]. Thus, people with sub-optimal exercise motives should be targeted with need-supportive strategies to optimise the quality of their motivation. Given the quality of motivation is predictive of whether an individual will engage with and sustain exercise behaviours [19], healthcare professionals should routinely discuss exercise motivations with patients and implement tailored need-supportive strategies.

Participants identified the presence or absence of need-supportive techniques in their social and healthcare context (i.e. provision of choice, a meaningful rationale, structure and guidance, and showing genuine interest and encouragement). Consistent with SDT, they noted several beneficial need-supportive techniques that healthcare professionals could adopt to promote more autonomous motivation. Participants particularly valued having control over the ‘what’, ‘when’, and ‘where’ of their exercise program, underscoring the importance of flexibility and a personalised program designed to enhance motivation. They also expressed a desire to understand the ‘why’ of exercise, noting that discussions about exercise were brief and lacked explanation of its benefits. This suggests that healthcare professionals discussing the benefits of exercise, particularly for ADT-related side effects, are missing opportunities to bolster motivation. Participants placed high importance on ‘how’ their exercise interactions occurred, which aligns with previous research indicating people receiving ADT value a sense of connection with their exercise therapist [34]. In addition to the need-supportive strategies reported in this study, previous research has identified additional techniques to foster motivation [19, 21, 26, 35, 36]. Teixeira et al. [36] described 21 SDT-based motivation and behaviour change techniques including exploring life aspirations (supporting autonomy), addressing obstacles for change (supporting competence), and encouraging asking of questions (supporting relatedness). Encouraging healthcare and exercise professionals to maximise their use of these need-supportive strategies, and minimise need-thwarting strategies, will create environments in which optimal exercise motivation can flourish.

Importantly, need-supportive strategies should not be used in isolation, but rather combined to optimise motivation [26]. A healthcare professional, for example, could offer an individual the choice of a home- or gym-based exercise program (autonomy support), while also helping them set a realistic weekly exercise target (competence support), in an empathetic and caring manner (relatedness support). However, it is unrealistic to expect healthcare professionals to effectively implement SDT-based need-supportive strategies without a solid understanding of the theory. Providing SDT-based training for clinicians has been shown to foster need-supportive exercise environments [35]. Such education must ensure healthcare professionals understand the value and evidence underpinning these techniques and feel confident in applying them in practice.

Although many participants reported more autonomous forms of motivation, many were still not meeting the recommended physical activity guidelines [11]. Given the median age of 74 years in this study, this population may face challenges adhering to these guidelines. Further research is needed to determine whether the current exercise recommendations are both feasible and essential for people with prostate cancer on ADT. For some, more flexible messages such as ‘something is better than nothing’ or ‘keep as active as current abilities allow’ may be more meaningful and achievable [37].

This study provides new insights into exercise motivation in people receiving ADT, but has some limitations. The sample was relatively homogeneous, consisting mainly of highly educated participants from a single metropolitan hospital. There may have been clinician-bias in referring patients and response-bias, with those opting to participate having a pre-existing interest in exercise. It is unclear how well the identified themes can be generalised to broader socio-demographic populations. The interview guide included broad questions about motivation and exercise rather than being structured around the theoretical concepts of SDT. Interviews structured around SDT concepts may have allowed a more in-depth analysis of exercise motivation. Finally, all men included in this study were receiving ADT, precluding exploration of whether motivations to exercise differ between those receiving and not receiving ADT, as well as how ADT may influence motivation. Although not explored in the present study, it is plausible that the debilitating side effects of ADT may also influence exercise motivation directly by impairing energy and perceived competence to engage in exercise.

## Conclusion

Guided by SDT, we identified reasons underlying why people with prostate cancer on ADT are motivated to exercise and factors within their social and healthcare context influencing their motivation. As exercise engagement depends on an individuals’ motivation, healthcare and exercise professionals should focus on the quality of this motivation and use need-supportive strategies to help people with prostate cancer on ADT to engage in and sustain exercise.

## Supplementary Information

Below is the link to the electronic supplementary material.

Supplementary file1 (PDF 240 KB)

Supplementary file2 (PDF 120 KB)

## Data Availability

Data are available from the corresponding author upon reasonable request.
