# Supplementary material for: “I've got plenty of energy when I’m doing something I want to do”: applying self-determination theory to exercise motivation in people with prostate cancer
Source: Support Care Cancer. 2025 Apr 3;33(4):347. doi: 10.1007/s00520-025-09410-z (PMC11968458; doi:10.1007/s00520-025-09410-z)
Supplement: Supplementary file 1 — Supplementary file1 (PDF 240 KB) [file 520_2025_9410_MOESM1_ESM.pdf]

**Interview guide**

**Optimising exercise engagement in men with high risk prostate cancer treated with androgen deprivation and radiation therapy (OECAD)**

## Protocol:

Qualitative interviews with participants will follow a semi-structured interview guide exploring:

- Exercise behaviours engaged in
- Perceptions of exercise
- Challenges to engaging in and/or maintaining exercise
- Engagement with structured exercise programs
- Perceptions regarding healthcare professionals advising about exercise.

## General introduction

- Welcome the participant
- Emphasize the importance of participation + encourage to share all ideas, opinions and personal experiences as well as concerns and suggestions. There are no right or wrong answers. It is all about your own experiences and ideas. During the interview you do not need to worry about what other participants may answer or think. We are interested in your own views and opinions on the topics we will discuss.
- The interview will be recorded, and some notes will be taking during the interview. We anticipate the interview will take between 15 – 40 minutes of your time.
- Do you have some questions before we begin? No, then the recorder will be turned on from now.

## Introduction question (patient)

- As you might remember we are talking with men, like yourself, who are getting hormone treatment for prostate cancer. How have things been for you since you started hormone treatment?
    - Probe:
        - Have your symptoms changed?
        - Do you feel like the treatment is causing side-effects?

## Transition question (patient)

- How does the treatment and the symptoms affect the things you do day to day?
    - Probe:
        - Are there things you have stopped doing?
        - Are there things you have started doing again?
        - How do you feel about these changes?
        - Does this impact on your health and well-being? *(in case not covered by 'feel about these changes').*

## Main questions (patient)

### 1. Can you tell me about your current physical activity?

**Attitude**

- How has your physical activity level changed since:
    - your diagnosis
        - Follow-up question: (why?)
    - start of hormone treatment?

            - Follow-up question: (why?)
        - In case answer is more about sedentary behaviour
            - How do you associate your sedentary behaviour with physical activity?
        - How do you feel about these changes?
- How has COVID19 impacted on your physical activity?
    - Did your routine activity change?
    - In what ways has COVID changed your physical activity?
    - Can you tell me how it has made physical activity more or less difficult?

**Awareness/knowledge**

- What would you need to be doing to be sufficiently physically active?
    - Why do you think that? (probe around guidelines)
    - What do you perceive as being sufficiently physically active?
    - Follow-up question (in case the original answer is to general): can you be more specific in terms of frequency and duration?
- How do you feel about your current level of physical activity?
    - Probe:
        - Would you like to be more active?
        - Do you feel you spend too much time sitting?
- Are you aware of any benefits of being physically active?
    - Probe:
        - General health benefits?
        - Cancer/ADT specific benefits?
- Are you aware of any risks from being physically active?
    - Probe:
        - General risks? – injury, etc
        - Cancer/ADT specific risks?
- A part of **physical activity** is **exercise**, what do you understand is meant by exercise?
    - *Emphasis the words in bold*
- Do you think there is a difference if your cancer doctor tells you to increase your **physical activity** compared to increase your **exercise**?
    - *Emphasis the words in bold.*

2. Some people find it challenging to be physically active, can you tell me about your motivation to be physically active?

**Enablers:**

- What would encourage you to be physically active?

  (Possible factors: additional comorbidities, side effects, peer support, environment, advice, technology) *preferable no examples would be given to participant.*

  *Reminder: Asking about the argument* ("Why do you think this factor would encourage you").
- How could healthcare professionals help you in engaging in exercise?
    - Who in your healthcare team is most likely to influence your physical activity level?
    - Follow-up question; why this person in specific?
- COVID: Did you access any services or resources to support activity?
    - How did you find them? (as in were they helpful or not?)

**Social support**

- Is there someone else important in offering support to engage in physical activity?
    - Why are they important?
    - How do they feel about you being physically active?
    - How do they influence your physical activity levels?

**Barriers**

- What makes physical activity difficult for you?
    - Has this changed since your cancer diagnosis and/or start of treatment?

      *Reminder: Asking about the argument* ("Why would this discourage you").

**Self-efficacy**

- How confident do you feel in your ability to be physically active?
    - Probe:
        - How might professional support and encouragement impact your confidence?
- Have you ever discussed engaging in physical activity with a healthcare provider?
    - Probe:
        - and who specifically?

- What could healthcare professionals do to best support you to exercise?
    - Probe:
        - What type of information about physical activity would be beneficial?
        - How should this information be communicated?

**Mode of delivery (cues to action)**

- What type of exercise would you be interested in/do you see yourself doing?
    - Probe:
        - Type of exercise?
        - Who with? (alone, peers, partner, friends, general public, ….)
        - Location? (hospital, home, local gym, community center, no preference, …)
        - In consultation with exercise professional?
        - How far are you willing to travel?
- What do you think about following a structured exercise program?
- Do you intend to enrol in the free/subsidised structured exercise program your Doctor discussed with you?
    - Follow-up question: (why (not)?)
- COVID19 and physical activity
        - What sort of services/resources would you like access to?
        - What would dis/encourage you to be physically active?

## COVID-19

- What has been your experience of cancer care during COVID-19?
    - Probe:
        - Has your care changed?
        - Have you had any changes or delays to treatment/appointments?
        - How did you feel about these changes?
- What has been your experience of other medical care such as GP?
    - Probe:
        - Has your care changed?
        - Have you had any changes or delays to treatment/appointments?
        - How did you feel about these changes?

## If participation in Man Plan/PCFA is still ongoing

- Up until today, how do you find the program?
  - Probe:
    - Do you notice any changes in yourself? (probe around this changes)
- Up until today, did you attend all of the sessions?
  - Probe:
    - When do you find it especially difficult to participate in the program?
    - What helps you in having a high participation rate?

## If completed participation in Man Plan/PCFA

- How did you find the program?
  - Probe:
    - Did you notice any changes in yourself? (probe around this changes)
- Did you attend all of the sessions?
  - Probe:
    - When did you find it especially difficult to participate in the program?
    - What helped you in having a high participation rate?
- How did this change your physical activity outside of the program?
- What type of exercise do you plan to do now that you're finished?
  - Probe:
    - Is there anything that could support you to do this?
    - Is there something that could it make difficult for you to keep going?

## Final questions

- (Interviewer summarizes) Does that sound about right?
- Are there other things you want to add about the topic of physical activity and exercising?

## End

- Do you have some questions you would like to ask?

- Thank you for the effort and time you have set aside to participate in this conversation, I really appreciate it
